# Clonal Population of Flucytosine-Resistant *Candida tropicalis* from Blood Cultures, Paris, France

**DOI:** 10.3201/eid1404.071083

**Published:** 2008-04

**Authors:** Marie Desnos-Ollivier, Stéphane Bretagne, Claire Bernède, Vincent Robert, Dorothée Raoux, Elisabeth Chachaty, Elisabeth Forget, Claire Lacroix, Françoise Dromer, the YEASTS group

**Affiliations:** * Institut Pasteur, Paris, France; † Hôpital Henri Mondor–Assistance Publique Hôpitaux de Paris, Créteil, France; ‡ Institut Pasteur Institut National de la Santé et de la Recherche Médicale Unité 657, Paris, France; § Centraal Bureau voor Schimmelcultures, Utrecht, the Netherlands; ¶ Institut Gustave-Roussy, Villejuif, France; # Hôpital Beaujon, Clichy, France; ** Hôpital Saint Louis, Paris, France

**Keywords:** *Candida tropicalis*, candidemia, flucytosine, antifungal drug resistance, polymorphic microsatellite markers, multilocus sequence typing, molecular typing, *URA3*, epidemiology, research

## Abstract

*Candida tropicalis* is a diploid ascomycetes yeast responsible for 4%–24% of candidemia. Resistance to flucytosine is rarely described for this species but was observed for 45 (35%) of 130 *C. tropicalis* isolates recovered from blood cultures in the Paris area in a 4-year survey. The aims of this study were to test the hypothesis that the flucytosine-resistant isolates could represent a subgroup and to determine the relationship between epidemiologic and genomic data. Epidemiologic data and gene sequences were analyzed, and molecular typing was performed. Our results suggest that a clone of flucytosine-resistant isolates, associated with malignancies and a lower mortality than that for other *C. tropicalis* isolates, is widespread in the Paris area. We propose the analysis of 2 polymorphic microsatellite markers coupled with *URA3* sequencing to track the clone.

## Abstract 2

Such isolates are widespread around clinical centers and are associated with malignancies that cause fewer deaths than other *C. tropicalis* isolates.

*Candida tropicalis* is a diploid ascomycetes yeast commonly found on the skin and in digestive tracts of healthy human hosts worldwide (*1*). Infections caused by *C. tropicalis* are reported in 4%–24% of patients with candidemia, depending on the country of study, underlying risk factors, and period of study (*2*). Primary resistance to flucytosine (5FC) occurs in <5% of all *Candida* species except for *C. krusei*, in which it is detected in up to 28% of isolates (*3*). It was thus unexpected to observe 35% resistance to 5FC among *C. tropicalis* isolates recovered from blood cultures in the active surveillance program on yeast-related fungemia implemented by the French National Reference Center for Mycoses and Antifungals (NRCMA) in the Paris area. The YEASTS program is designed to analyze the epidemiologic trends of yeast fungemia by collecting isolates and epidemiologic and clinical data. The second objective is to study the clinical isolates in terms of species, antifungal susceptibility profiles, and genetic diversity to look for associations between subtypes of isolates and epidemiologic/clinical parameters. To test the hypothesis that the 5FC resistant (_R_5FC) isolates could represent a different species or a subgroup, the _R_5FC and susceptible (_S_5FC) isolates were compared on the basis of several phenotypic and molecular features.

## Materials and Methods

### Strains

Clinical isolates of *C. tropicalis* recovered from blood cultures during the YEASTS program from October 1, 2002, through September 30, 2006, were selected for the study. Epidemiologic and clinical data concerning the patients were collected by using a standardized electronic form. Isolates (1 isolate/patient) were sent to NRCMA for identification and MIC determination (see below). All isolates were stored frozen in 40% glycerol at –80°C.

The type strain of *C. tropicalis* CBS 94 (ATCC 750, _S_5FC) was included in the study as a reference. In addition, 29 strains of taxonomic synonyms available at the Centraalbureau voor Schimmelcultures (CBS, Utrecht, the Netherlands) were studied.

### Phenotypic Characterization of All *C. tropicalis* Isolates

All isolates were identified at the species level by using the assimilation patterns obtained with the commercialized strips ID32C (bioMérieux, Marcy-l’Etoile, France). MICs to 9 systemic antifungal agents were determined for all clinical isolates and the type strain by using the EUCAST microdilution method (*4*). For nonclinical isolates, only MICs of 5FC and fluconazole were determined.

### Additional Studies on Selected Isolates

#### Growth Characteristics

For the first 16 _S_5FC and 14 _R_5FC consecutive isolates of *C. tropicalis* and for CBS 94 other studies were performed. Additional carbon sources were tested by using the commercial strips CH50 (bioMérieux). Maximal temperature of growth (42°C or 45°C) was determined on Sabouraud dextrose agar. Growth in hyperosmolar medium (50% glucose or 10% NaCl) was also evaluated.

#### Nucleotide Sequence Determination

After 24 hours of incubation at 27°C on Sabouraud agar plates, single colonies were discharged in 1 mL of distilled water in a microcentrifuge tube, and DNA extraction was performed by using the High Pure PCR Template Preparation Kit (Roche Applied Science, Mannheim, Germany) according to manufacturer’s instructions. Universal fungal primers were used for the amplification of the internal transcribed spacer 1 (ITS1)–5.8S-ITS2 (primers V9D and LS266 [*5*,*6*]) and the 26S (primers NL1 and NL4 [*7*]) rDNA regions. Primers (Table 1) were designed to amplify a partial sequence of the actin gene (GenBank accession nos. AJ389059 and AJ508499) and the 14-α-demethylase gene (GenBank accession nos. AY942646 and M23673). Reaction volumes of 20 μL contained 1 μL of genomic DNA, 1.25 U of AmpliTaq Gold, 2 μL of PCR buffer 10×, 2 μL of 25 mmol/L MgCl_2_ 2 μL of 2.5 mmol/L deoxyribonucleoside triphosphates (dNTPs) (Roche), and 1 μL (10 μM) of primers. The PCR products were amplified by using the ICycler Thermocycler (Bio-Rad, Marnes-La-Coquette, France) set up with a first cycle of denaturation for 10 min at 95°C, followed by 30 cycles of denaturation at 94°C for 30 s, 30 s at the relevant annealing temperature, elongation at 72°C for 30 s, and a final extension step of 10 min at 72°C. Both strands of purified amplified fragments were sequenced at the Genopole of the Pasteur Institute, on an ABI Prism 3700 DNA Analyzer (Applied Biosystems, Courtaboeuf, France), with the same primers that were used in the PCR step. Sequences were edited with Chromas Pro version 1.33 (Technelysium Pty Ltd, Helensvale, Queensland, Australia). Multiple sequences alignments were performed with Clustal W version 1.8 (www.ebi.ac.uk/clustalw/).

**Table 1:** Primers sequences and amplification parameters used in the present study

Locus	Primer	5′ labeling	Sequence (5′ → 3′)*	Annealing temperature, °C	
14αDM†	DMC1		>TGGGTGGTCAACATACTTC	50	
	DMC2‡		<CATCTRTGTCTACCACCACC		
Actin	ACTa		>AAGGTATTATGGTTGGTATGG	55	
	ACTb		<TCGAAATCTAAAGCAACGTAA		
URA3	URAF	HEX§	>ATTGGATAGTCCCTCTAAACTCACTACTA	55	
	URAr		<AGCATTAGTTATATCACTCCACGATGAA		
	URAF2		>TGCCGATATTGGAAATACAGTTA	50	
	URAr2		<AATCAACTATTCAAGTTGACCG		
	CTU2		<GTTGGAACATCAATTGATGCACATAAAT	55	
Unknown	CT14a	6FAM¶	>GTAAATCTTGTATACCGTGGA	55	
	CT14b		<TAGCCCATTTTCTAGTTTTGC		
FCY1	CTCDf		>ATCATTAGTTCAGATGGTAAAGTCTTG	58	
	CTCDr		<CCTTTTTAGTAACATGTCTATTCTCCA		
FCY2	CTCP1f		>TGCCCATAAATTAAATGCAGAA	58	
	CTCP1r		<GGAAGCAACAAACCCAAAAA		
FCY2	CTCP2f		>TGCTGCCGATTATGTTGTTT	58	
	CTCP2r		<GTGAAAACGAGCCAATCCAT		
FUR1	FUR1f		>TCATCAAAACCATGTCTGCTG	58
	FUR1r		<AAGTGTATGTAGTGATAATTGCTATGC	

*>, Sense primer; <, antisense primer. †14 α demethylase. ‡R = G or A. §4, 7, 2′,4′, 5′,7′-hexachloro-6-carboxyfluorescein. ¶6-carboxyfluorescein.

Following preliminary results, primers were then designed to amplify the complete sequence of the orotidine-5′-phosphate decarboxylase gene (URA3, GenBank accession no. AF040702) (Table 1). Furthermore, the complete sequences of *FCY1* (coding for the cytosine deaminase), *FCY2* (coding for the purine cytosine permease), and *FUR1* (coding for the uracil phosphoribosyl transferase) were determined. Primers were designed by using sequences from the Broad Institute *C. tropicalis* database genome (locus CTRG_02927.3 for *FCY1*, locus CTRG_02059.3 for *FCY2,* and locus CTRG_02689.3 for *FUR1*) (Table 1). The sequences were amplified as described above (except for the duration of annealing and elongation [1 min] when using the primers set CTCP1f/CTCP1r). The sequences were translated with the standard genetic code (www.bioinformatics.org/sms/index.html). The resulting protein sequences were aligned with the BioloMICS software (BioloMICS, version 7.2.5, BioAware S.A., Hannut, Belgium).

#### Microsatellite Selection

*C. tropicalis* genome sequences available from GenBank databases and from the Broad Institute (www.broad.mit.edu/annotation/fungi/candida_tropicalis) were studied to identify sequences containing microsatellite repeats. Two polymorphic microsatellite markers (PMMs) were selected, 1 upstream of the *URA3* gene (URA3 PMM) and 1 on a nonannotated sequence (CT14 PMM). Oligonucleotide primers were designed from the sequence of the corresponding flanking regions to obtain PCR products ranging in size from 100 bp to 200 bp. One primer of each set was 5′ labeled with different dyes (Table 1). PCR was conducted independently for the 2 loci in a 20-μL reaction volume containing 2 μL of extracted DNA, 1.25 U of AmpliTaq Gold, 2 μL of PCR Buffer 10×, 4 μL of 25 mmol/L MgCl_2_ , 2 μL of 2 mmol/L dNTPs, and 0.2 μL (10 μM) of primers. PCR amplifications were performed for a total of 27 cycles by using the following conditions: denaturation at 95°C for 30 s, annealing at 55°C for 30 s, extension at 72°C for 1 min, and a final extension step of 5 min at 72°C. Two microliters of each PCR product mixed with 20 μL of formamide and 0.5 μL of an internal standard labeled with 6-carboxy-X-rhodamine dye (GeneScan-500 Tamra, Applied Biosystems) was run on an ABI Prism 310 Genetic Analyzer (Applied Biosystems). Sizes of the allele and PCR fragments were determined with GenScan 3.0 (Applied Biosystems, Weiterstadt, Germany). To assign a specific length to a PCR fragment, all electromorphs were aligned with that of the type strain (CBS 94). Each allele was named after the length of PCR fragments. Isolates for which 1 signal was observed for a given locus on the electromorph were considered homozygous for this locus by analogy with what is reported for another diploid yeast, *C. albicans* (*8*).

#### Multilocus Sequence Typing Analysis

Three of the 6 MLST loci recently described were analyzed as reported in Tavanti et al. (*9*). These loci were selected because, according to these authors, they were associated with more polymorphism (XYR1 and SAPT4) and with antifungal resistance (MDR1). Both strands of purified amplified fragments were sequenced, and sequences were edited as described above. Heterozygosity was defined by the presence of 2 coincident peaks of similar height in the forward and the reverse sequence chromatograms. The 1-letter code for nucleotides from the nomenclature of the International Union of Pure and Applied Chemistry (IUPAC, www.bioinformatics.org/sms/iupac.html) was used. Sequences were compared with the allele sequences of the *C. tropicalis* MLST database (www.pubmlst.org/ctropicalis). For each gene, distinct alleles were identified and numbered by using the Internet-based MLST program (www.mlst.net). New alleles were submitted to the MLST *C. tropicalis* database.

### Statistical Analysis

In accordance with French regulations, the clinical database was approved by the Commission Nationale de l’Informatique et des Libertés. Information concerning demographic data, risk factors for candidiasis, and outcome 30 days after the diagnosis of fungemia were recorded. We considered 3 groups of patients according to the infecting isolate: _S_5FC, _R_5FC that belong to the clone (_R_5FC clone, see below), and _R_5FC that do not belong to the clone (termed “other _R_5FC”). The sociodemographic and clinical characteristics were compared between the 3 groups of isolates by using the Fisher exact test. The χ^2^ Armitage trend test (*10*) was used to assess a trend in the evolution of the _R_5FC clone’s proportion among resistant strains across years of study. Multinomial logistic regression (*11*) adjusted on clinical center was used to investigate the factors associated with infection by the _R_5FC clone or other _R_5FC isolates compared to _S_5FC isolates according to sociodemographic and clinical characteristics. A logistic regression model adjusted on clinical center was also performed to identify the factors associated with the acquisition of the _R_5FC clone compared with other _R_5FC isolates. Regression models were constructed by using the backward procedure. First, all covariates with a p value <0.25 in univariate models were simultaneously entered into the regression model. The set of covariates with the largest p value was iteratively removed from the model until all of the covariates (or blocks of covariates) remaining in the reduced model had a p value <0.05. Statistical analyses were performed with Stata software, version 9.0 (StataCorp, College Station, TX, USA).

## Results

### Phenotypic Characterization of _R_5FC Isolates

We analyzed the episodes of fungemia caused by *C. tropicalis* and recorded during the first 4 years of the YEASTS study; 130 episodes were recorded in 24 of the 27 participating centers. Distribution of flucytosine MICs showed 2 populations, 1 with MICs <2 μg/mL and 1 with MICs >8 μg/mL (Figure). In light of these results, susceptibility to 5FC (_S_5FC) was defined by an MIC <8 μg/mL and resistance (_R_5FC) by an MIC >8 μg/mL.

**Figure:**
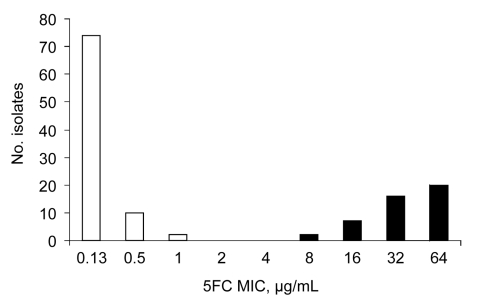
Distribution of 130 *Candida tropicalis* isolates recovered from blood cultures during the first 4 years of an active surveillance program (YEASTS study) on yeasts fungemia in the Paris area, France (October 2002 through September 2006), according to the MICs of flucytosine determined with the EUCAST microdilution method (*4*).

The proportion of _R_5FC isolates (45 [35%]) of the 130 isolates) was uneven, ranging from 0% to 67% of the isolates, depending on the center of isolation. However, the proportion of _R_5FC isolates did not differ over the study period (data not shown). We first studied the characteristics of a subset of 16 _S_5FC and 14 _R_5FC *C. tropicalis* isolates (strain CBS 94 had all the characteristics of _S_5FC clinical isolates described below but is not included in the analysis). There was no difference in terms of growth in hyperosmolar media between _R_5FC and _S_5FC clinical isolates. By contrast, _R_5FC and _S_5FC isolates differed in the proportion of isolates growing at 45°C (40% vs. 100%, p<0.001) and assimilating starch (12.5% vs. 50%, p = 0.054) and xylitol (62.5% vs. 12.5%, p = 0.009). No difference in the MIC of azoles or caspofungin was noted. All 29 strains of *C. tropicalis* synonyms stored at the CBS exhibited 5FC MICs <0.5 μg/mL.

### Genotypic Characterization of _R_5FC Isolates

This subset of isolates (16 _S_5FC and 14 _R_5FC) was further analyzed. The deletion of 1 nucleotide (A) in position 106 (according to the type strain sequence, GenBank accession no. AY939810) of the ITS2 region was observed in 14 (100%) of the 14 _R_5FC isolates compared to 5 (32%) of the 16 _S_5FC isolates (p<0.001) (GenBank accession no. EU288196). No difference in nucleotide sequences was found for the D1/D2 region of the 26S rDNA or in the portion of the 14-α demethylase (490 bp) and actin (550 bp) genes analyzed. PMM results are summarized in Table 2. The URA3 and CT14 PMM led to 6 and 7 different allelic associations, respectively. The association of both markers led to 13 PMM profiles. The 14 _R_5FC isolates had the same URA3/CT14 PMM profile; however, among the 16 _S_5FC, 15 had a PMM profile different from that of the _R_5FC, and 1 (ODL6–560) had the _R_5FC PMM profile. All but 1 (ODL2–237) of the _R_5FC isolates had the same MLST profile, whereas none of the _S_5FC isolates exhibited the same combination of the 3 MLST studied. The entire *URA3* nucleotidic sequence of the translated region was identical except in 1 position (GenBank accession no. EU288194 for the type strain CBS 94 and EU288195 for 1 of the _R_5FC isolates). _S_5FC isolates were either homozygous or heterozygous at position 529 (A-A or A-G), whereas all the _R_5FC isolates were homozygous G-G. This produced a change of K177E (lysine → glutamate) for the _R_5FC isolates.

**Table 2:** Comprehensive analysis of 30 *Candida tropicalis* isolates*

Strain no.	5FC MIC (μg/mL)	Month of isolation	SNP ITS2	PMM alleles			MLST			*URA3* base 529
				URA3	CT14		MDR1	XYR1	SAPT4	
ODL1–18	>64	2002 Nov	–	178/178	148/151		20	26	10	G
ODL1–40	>64	2002 Nov	–	178/178	148/151		20	26	10	G
ODL1–41	>64	2002 Nov	–	178/178	148/151		20	26	10	G
ODL1–53	16	2002 Dec	–	178/178	148/151		20	26	10	G
ODL2–105	>64	2003 Mar	–	178/178	148/151		20	26	10	G
ODL2–198	32	2003 Jul	–	178/178	148/151		20	26	10	G
ODL2–199	>64	2003 Jul	–	178/178	148/151		20	26	10	G
ODL3–237	>64	2003 Sep	–	178/178	148/151		18	26	10	G
ODL3–293	>64	2003 Oct	–	178/178	148/151		20	26	10	G
ODL4–311	>64	2003 Sep	–	178/178	148/151		20	26	10	G
ODL4–328	>64	2003 Nov	–	178/178	148/151		20	26	10	G
ODL4–341	>64	2003 Nov	–	178/178	148/151		20	26	10	G
ODL5–426	>64	2003 Dec	–	178/178	148/151		20	26	10	G
ODL6–558	32	2004 Apr	–	178/178	148/151		20	26	10	G
ODL1–58	<0.125	2003 Jan	+	176/176	148/148		24	30	7	A
ODL3–211	0.25	2003 Jul	+	174/178	148/148		1	**79**	1	A-G†
ODL3–231	<0.125	2003 Sep	–	176/176	151/151		**66**	9	18	A
ODL4–302	0.25	2003 Sep	–	176/178	148/151		4	36	23	A
ODL4–347	0.25	2003 Nov	+	174/174	151/154		7	52	6	A
ODL4–384	0.5	2003 Dec	–	174/176	151/151		**67**	4	19	A
ODL5–460	<0.125	2004 Feb	+	174/178	148/148		**68**	**76**	36	A-G†
ODL5–474	<0.125	2004 Mar	+	174/174	154/154		7	52	6	A
ODL5–476	<0.125	2004 Mar	+	178/178	151/151		27	4	11	A
ODL5–485	<0.125	2004 Mar	–	176/178	148/157		22	41	7	A
ODL5–488	<0.125	2004 Apr	+	176/178	148/151		25	24	7	A
ODL6–504	<0.125	2004 Feb	+	174/178	148/151		22	9	38	A
ODL6–511	<0.125	2004 Apr	+	174/178	148/148		1	**80**	1	A-G†
ODL6–521	<0.125	2004 May	+	176/178	148/151		**69**	**77**	**41**	A
ODL6–539	<0.125	2004 Mar	+	174/174	148/154		58	48	13	A
ODL6–560	<0.125	2004 Jul	–	178/178	148/151		4	36	23	A
CBS94	<0.125	–	+	176/176	148/148		**70**	**78**	5	A

*5FC, flucytosine; SNP, single nucleotide polymorphism; ITS2, internal transcribed spacer 2; PMM, polymorphic microsatellites marker; MLST, multilocus sequence typing (**boldface** corresponds to new alleles). †A-G heterozygous.

Thus, results obtained with nucleotide changes (deletion of A in position 106 in the ITS2 region, mutation in position 529 of the *URA3* gene) and polymorphisms in 3 MLST and 2 PMMs suggested that the 14 _R_5FC studied were clonal. We thus decided to use the 2 PMMs (URA3 PMM, CT14 PMM) to genotype all the *C. tropicalis* isolates recovered during the study period in the YEASTS program. Of the 130 *C. tropicalis* isolates (including the 30 isolates studied above), 45 were _R_5FC (Table 3). Thirty-three different profiles were observed when both PMMs were combined for the 130 isolates. Among the 45 _R_5FC, a total of 29 isolates exhibited the profile associated with the _R_5FC clone; 16 were different, with 11 different profiles, 6 of which were shared with _S_5FC isolates. Among the _S_5FC isolates, 4 had the PMM profile associated with the _R_5FC clone. The *URA3* gene was sequenced for these 4 isolates, and none had a G in position 529.

**Table 3:** Distribution of the polymorphic microsatellites markers (PMM) profiles among 130 *Candida tropicalis* isolates, according to their susceptibility to flucytosine (5FC)*

Allelic association		Total no. isolates (N = 130)	No. isolate types		
URA3 PMM	CT14 PMM		_S_5FC (n = 85)	_R_5FC clone (n = 29)	Other _R_5FC (n = 16)
172/172	142/148	3	3	–	–
172/172	148/154	2	2	–	–
172/174	142/148	3	2	–	1
172/174	148/148	1	1	–	–
172/174	148/154	1	1	–	–
172/176	142/148	5	4	–	1
174/174	142/148	5	3	–	2
174/174	148/148	1	–	–	1
174/174	148/151	1	1	–	–
174/174	148/154	6	6	–	–
174/174	151/154	1	1	–	–
174/174	154/154	1	1	–	–
174/176	142/148	6	6	–	–
174/176	148/151	1	1	–	–
174/176	151/151	3	3	–	–
174/178	142/148	8	5	–	3
174/178	145/151	1	–	–	1
174/178	148/148	5	5	–	–
174/178	148/151	2	1	–	1
176/176	142/148	11	10	–	1
176/176	148/148	3	3	–	–
176/176	148/151	4	4	–	–
176/176	151/151	1	1	–	–
176/178	142/148	3	3	–	–
176/178	145/151	1	1	–	–
176/178	148/148	1	–	–	1
176/178	148/151	5	5	–	–
176/178	148/157	1	1	–	–
176/180	151/151	1	1	–	–
178/178	142/148	3	2	–	1
178/178	145/151	6	3	–	3
178/178	148/151	33	4	29	–
178/178	151/151	1	1	–	–

*S subscript, susceptible; R subscript, resistant.

We then studied genes potentially involved in the mechanisms of 5FC resistance. For the FUR1 sequences, no missense mutation was observed, and the complete coding sequence of the type strain CBS 94 (GenBank accession no. EU327978) was similar to the sequence of the _R_5FC clone; however, a few silent mutations were observed in a few _S_5FC isolates (GenBank accession nos. EU327979, EU327980, and EU327981). Concerning the cytosine deaminase sequences (FCY1), only 1 silent mutation, C21T, occurred for the _R_5FC clone (GenBank accession no. EU327982). Finally, for the purine cytosine permease (FCY2), the sequences of the type strain (GenBank accession no. EU327983) and of the _R_5FC clone were similar. A few heterozygosities were observed for the _S_5FC isolates (GenBank accession nos. EU327984 and EU327985), but all these mutations were silent.

### Factors Associated with Fungemia Caused by the *C. tropicalis*_R_5FC Clone

All 130 isolates corresponded to incident fungemia in different persons. The _R_5FC clone was recovered during the 4 years of study with a trend toward a decreased proportion over time (11/13 [85%], 6/10 [60%], 8/13 [61.5%], and 4/9 [44%]) during the first, second, third, and fourth year of the study, respectively; p = 0.06). The proportion of the clone also varied across clinical centers (data not shown). Factors associated with fungemia caused by the _R_5FC clone of *C. tropicalis* were analyzed (Table 4). The proportion of patients infected by the _R_5FC clone was significantly higher among patients with malignancies but their death rate was significantly lower than for patients infection with other _R_5FC or _S_5FC isolates. Multinomial logistic regression was adjusted by clinical centers to investigate the factors associated with infection by the _R_5FC clone or others _R_5FC isolates compared with _S_5FC isolates. The risk of being infected by the _R_5FC clone compared with a _S_5FC isolate significantly increased in case of malignancy (odds ratio [OR] 3.7, 95% confidence interval [CI] 1.4–10.1, p = 0.009), and the risk for death at day 30 after fungemia was significantly decreased in patients infected by the _R_5FC clone compared with the _S_5FC isolates (OR 0.3, CI 0.1–0.9, p = 0.04), while no independent factor accounted for infection by other _R_5FC isolates versus an _S_5FC isolate. The only independent factor associated with infection by the _R_5FC clone compared with other _R_5FC isolates was the death rate at day 30 (OR 0.1, CI 0.03–0.6, p = 0.006).

**Table 4:** Patient characteristics according to the 3 categories delineated by the susceptibility of the *Candida tropicalis* isolates to flucytosine (5FC) and their belonging to the _R_5FC clone*

Characteristic	_S_5FC (n = 85)	_R_5FC clone (n = 29)	Other _R_5FC (n = 16)	p value†
>60 y of age	39 (46)	17 (59)	9 (56)	0.452
Male	55 (65)	18 (62)	10 (63)	0.962
Had malignancies	41 (48)	22 (76)	9 (56)	0.033
Cancerous	15 (18)	7 (24)	5 (31)	0.374
Hematologic	26 (31)	15 (52)	4 (25)	0.082
In intensive care unit	44 (52)	12 (41)	4 (25)	0.116
Had central venous catheter	68 (80)	24 (83)	12 (75)	0.894
Had recent surgery	27 (32)	8 (30)	4 (25)	0.918
Had prior antifungal therapy	11 (13)	0	2 (13)	0.093
Died before day 30	34/81 (42)	6/28 (21)	10/15 (67)	0.014

*S subscript, susceptible; R subscript, resistant. Values are no. (%). †Fisher exact test.

## Discussion

The bimodal distribution of 5FC MICs against *C. tropicalis* isolates prospectively collected from 27 different clinical centers in the Paris area (YEASTS program, Figure) suggested that the *C. tropicalis* population was heterogenous. On the basis of physiologic characteristics and molecular analysis (nucleotide sequences of the ITS regions, D1/D2 region of the large subunit, and large portions of the actin and 14-α-demethylase genes showing >99% similarity), we first assessed a subset of isolates (the first consecutive 14 _R_5FC and 16 _S_5FC isolates) and determined that both populations belong to the same species.

All 14 _R_5FC isolates had a single nucleotide deletion in position 106 of the ITS2 region, although 5 of the 16 _S_5FC isolates harbored it. When additional genotypic markers were used, all 14 _R_5FC isolates had the same allelic combination for 2 PMMs selected (URA3 and CT14), the same missense mutation in the *URA3* gene, and the same diploid sequences for the 3 MLST loci studied. By contrast, only 1 of 16 _S_5FC isolates had the same PMM profiles as the _R_5FC isolates, but this isolate differed in its MLST profile and the lack of mutation in the *URA3* gene. In addition, the 16 _S_5FC isolates exhibited 16 different MLST and 12 different PMM profiles. This finding suggested the existence of a _R_5FC clone, but the rest of the population was genetically diverse. We thus analyzed the 130 isolates of *C. tropicalis* collected over 4 years in the YEASTS program by using the 2 PMMs and sequenced the *URA3* gene when the PMM profile was identical to that of the 14 _R_5FC isolates previously studied. We discovered that 29 (64%) of 45 _R_5FC isolates had an identical PMM profile (_R_5FC clone), while 11 and 30 different PMM profiles were found among the other 16 _R_5FC isolates and the 85 _S_5FC isolates, respectively. According to these data, we assumed that these 29 _R_5FC isolates were clonal or at least highly genetically related.

The proportion of 35% of *C. tropicalis* isolates resistant to 5FC is unusual (*3*). Other studies report between 0 (*12*) and 15% (*13*) with intermediate values (*14*–*16*), and all the isolates stored as *C. tropicalis* in the CBS collection since 1912 exhibited 5FC MIC <0.5 μg/mL. When we started the YEASTS program in 2002, the proportion of _R_5FC was already at 46% and the clone accounted for 85% of the _R_5FC isolates. The trend test suggested that the dispersal of the clone is declining in the Paris area. Whether this decline is specific for blood isolates or is a geographically and temporally restricted phenomenon deserves evaluation by using isolates collected over time from various body sites and geographic areas. An old report on isolates collected from various regions of France established with a nonstandardized technique that as many as 70% of the 63 isolates tested had an MIC >32 μg/mL in the 1980s (*17*), and a recent study from Germany on clinical isolates recovered from various body sites including blood reported that 58.3% of isolates were resistant (*18*). Whether any of these isolates belong to the _R_5FC clone would be of interest. Of note, a recent study of 104 *C. tropicalis* clinical isolates recovered from various countries (*9*) showed that none of the 5 _R_5FC isolates collected in the United Kingdom has the MLST profile of the _R_5FC clone.

In the univariate analysis, patients infected by the _S_5FC or _R_5FC isolates or by the _R_5FC clone differed significantly in terms of proportion of underlying malignancies (higher in patients with the clone) and death rate 30 days after fungemia (lower for patients infected by the clone). The _R_5FC isolates as a whole, and the _R_5FC clone specifically, were unevenly distributed around the Paris area. When adjusted for clinical center, logistic regression analysis showed that, compared to infection by _S_5FC isolates, no factor was independently associated with infection by _R_5FC isolates other than the clone, whereas 2 parameters were associated with infection by the clone. Indeed, malignancies multiplied the risk of being infected by the clone by almost 4 and the risk for death was divided by 3 in case of infection with the clone. *C. tropicalis* fungemia, independent of susceptibility to flucytosine, has already been associated with hematologic malignancies (*19**,**20*) (unpub. data from the YEASTS group). Whether the _R_5FC clone is less virulent, as established for *C. albicans* isolates with decreased susceptibility to 5FC, remains to be determined (*21*).

The resistance to 5FC was associated with the K177E mutation in the *URA3* gene in the clone. The mechanism of 5FC action is a consequence of intrafungal formation of 2 metabolites, 5-fluorodeoxyuridine monophosphate and 5-fluorouridine triphosphate, which alter DNA and protein synthesis (*22*). The URA3 enzyme (orotidine 5′-phosphate decarboxylase, ODCase) is involved in the metabolic pathway of uridyl-monophosphate (UMP), which is a substrate of thymidylate synthetase and UMP kinase, both involved in nucleic acid synthesis. This mutation involves an amino acid already known to be variable among reference strains (e.g., ATCC 20336), but it has not been associated with modification of the URA3 properties thus far (T. Noël, pers. comm.). Nevertheless, this mutation could, for example, modify the tridimensional structure of the protein, thereby affecting the binding affinity of the substrate for the catalytic site and thus modifying the ODCase efficacy. The ODCase is not known to interfere directly with 5FC activity. However, one of the resistance mechanisms against 5FC consists in increasing the transcription of all the genes involved in the de novo pyrimidine biosynthetic pathway (including *URA3*) to overproduce UMP (*23*).

The K177E mutation is associated with a specific PMM upstream the gene, with a possible role in the level of transcription. The fact that this PMM is homozygous may be due to a loss of heterozygosity. This phenomenon has been recently reported for *C. albicans* and the resistance to azoles (*24*) and for a specific *C. albicans* isolate and the resistance to caspofungin (*25*).

The mutations described in the 5FC resistance of *C. albicans* (*26*) or *C. lusitaniae* (*27*) involved 3 major genes: *FCY2* coding for the purine cytosine permease, which enables 5FC to enter the fungal cell; *FCY1* coding for the cytosine deaminase, which transforms 5FC into 5FU; and *FUR1* coding for the uracil phosphoribosyl transferase, which transforms 5FU into 5FUMP. The fact that the clone was susceptible to 5FU suggests that the 5FC resistance could result from a mutation in the cytosine deaminase, the cytosine permease, or both (*23*). However, the sequences of the _R_5FC clone, some _S_5FC isolates, and the type strain CBS94 did not show any mutation in coding sequences of *FCY1*, *FCY2,* or *FUR1* susceptible to explain the resistance of the clone to 5FC. The mechanism explaining the possible relationship between the specific PMM (URA3 178/178 and CT14 148/151), the K177E mutation, and the resistance to 5FC remains to be determined.

Our results suggest that a clone of _R_5FC isolates responsible for fungemia is widespread among patients hospitalized with malignancies in the Paris area and is associated with a lower mortality than that of other *C. tropicalis* isolates. Despite a trend toward a decreased proportion over time, further studies are needed to assess this clone’s geographic and temporal distribution. Analysis of the 2 PMMs described in this study, coupled with determination of nucleotide at position 529 in the *URA3* gene, should provide reliable means to track this clone.
